# Creating the perfect plasmonic wave

**DOI:** 10.1038/s41377-022-00926-1

**Published:** 2022-07-22

**Authors:** Günter Steinmeyer

**Affiliations:** 1 Max Born Institute for Nonlinear Optics and Short Pulse Spectroscopy, Berlin, Germany; 2 Institute of Physics, Humboldt University, Berlin, Germany

**Keywords:** Nanophotonics and plasmonics, Nanophotonics and plasmonics

## Abstract

Exploiting a plasmonic resonance, near-perfect grating structures have been reported, with a regularity that exceeds typical commercially available diffraction gratings.

## Abstract (web summary)

Plasmonics can help to produce optical gratings with surprisingly high regularity and writing speed, surpassing mechanically ruled gratings in both aspects.

## Graphical abstract

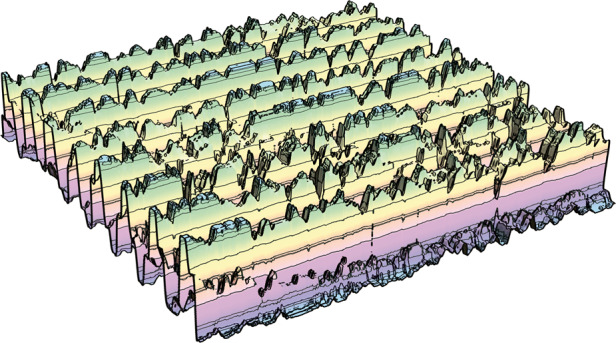

Diffraction gratings led the path towards high-resolution spectroscopy in the early 20th century, providing vital experimental information that strongly contributed to our current understanding, for example, of atomic spectra or the expanding universe. As early as 1900, Albert Michelson understood the need for reliably manufacturing large gratings in order to obtain resolving powers of 500,000 or more^1^. He originally considered a period of 5 years sufficient to build a ruling engine that would allow about 10 inches of travel^1,2^. The mechanical key component in such a ruling engine is the lead screw; and this component turned out to be much more problematic than originally anticipated, that is, ruling groove after groove, the lead screw needs to ensure that deviations from the perfect periodicity remain within one tenth of the grating period. Such accuracy was at least an order of magnitude beyond typical capabilities of the time. Frustrated by the slow progress of his assistants, Michelson eventually decided to take things into his own hands. During an absence of his assistants, he finished the remaining section of the lead screw himself^1^. Unfortunately, this sudden haste spoiled the final 2 inches of his ruling engine. This issue would only be fixed more than 20 years after Michelson’s death, introducing a feedback mechanism that, quite ironically, relied on an interferometer. Nevertheless, the ruling engine was still good enough to produce an 8-inch grating, which he would proudly take with him to his Nobel Laureate ceremony in Stockholm in 1907, however, only to get accidentally dropped, destroying any evidence for its projected resolving power^2^.

Switching from early tragedy to modern times, high-resolution spectroscopy has advanced to comb-based methods, often making the use of gratings obsolete. Nevertheless, there is still considerable interest in large-area gratings, which is mostly motivated by chirped-pulse amplification schemes currently progressing towards the generation of tens of petawatts of peak power;^3^ and suitable compression gratings are still limited to meter size dimensions. The work of Geng et al. now offers an interesting new path towards fabrication of the perfect grating^4^. Rather than externally controlling mechanical ruling, the authors effectively rely on a plasmonic standard, that is, exploiting ablation/oxidation cycles in a metal coated substrate, they exploit a sharp resonance of surface-plasmon polaritons that leads to reproducible formation of a grating structure with well-controlled ~900 nm spacing. In the laser materials community, this mechanism of grating formation is known as laser-induced periodic surface structures^5^ (LIPSS), which, however, are infamously known for their irregularity and would not satisfy even the lowest standards for spectroscopic gratings. Analyzing the regularity of Geng et al.’s grating structures^4^ and comparing with published examples in the literature^1,6–8^, one observes that the grating period varies only by 1.5% in a grating produced with 0.2 mm/s writing speed, see Fig. 1. At 100 times larger speeds, the irregularity increases to 4%, which is still in the acceptable range for commercial ruled gratings^1^.

**Fig. 1:**
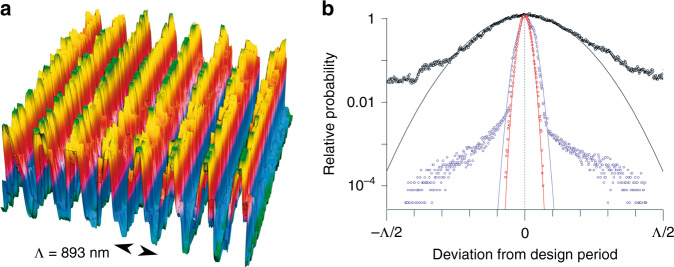
Grating regularity. **a** 3D visualization of a small-scale cut out of Fig. 2a of Ref. 3. **b** Extracting the grating phase from this figure via the Hilbert transform^8^, one can evaluate deviations from perfections, which is shown in terms of relative probabilities vs. deviation in units grating period (red symbols). For comparison, the identical analysis was run for two commercial replicated ruled gratings (black^6^, blue^7^). Parabolic fits (curves) indicate the prevalent character of short-term noise excursions; deviations from these fits are indicative of slower drift-like deviations from the desired grating period

While it certainly remains to be seen whether plasmonic standards can really out-compete interferometric ones and whether this approach proves scalable toward meter sizes, the approach of Weng et al. opens a new avenue for manufacturing diffraction gratings with surprisingly good precision. Let’s stay optimistic like Michelson did and be confident that we will see such progress in a few years to come.
